# HRD1 sensitizes breast cancer cells to Tamoxifen by promoting S100A8 degradation

**DOI:** 10.18632/oncotarget.15797

**Published:** 2017-03-01

**Authors:** YanYang Wang, AiBin Guo, XiuBin Liang, Min Li, Ming Shi, Yan Li, Gareth Jenkins, XiaWen Lin, XueFei Wei, ZhiJun Jia, XueFeng Feng, DongMing Su, WanHua Guo

**Affiliations:** ^1^ Department of Nuclear Medicine, The Affiliated Drum Tower Hospital of Nanjing University, Nanjing, China; ^2^ Department of Geriatric Medicine, The Affiliated Drum Tower Hospital of Nanjing University, Nanjing, China; ^3^ Department of Surgical Oncology, The First Affiliated Hospital With Nanjing Medical University, Nanjing, China; ^4^ Center of Pathology and Clinical Laboratory, Sir Run Run Hospital, Nanjing Medical University, Nanjing, China; ^5^ Institute of Advanced Materials, Nanjing University of Post and Telecommunication, Nanjing, China

**Keywords:** Tamoxifen, HRD1, S100A8, chemotherapy resistance

## Abstract

Estrogen receptor alpha positive (ER+) of breast cancer could develop resistance to antiestrogens including Tamoxifen. Our previous study showed that the E3 ubiquitin ligase HRD1 played an important role in anti-breast cancer. However, its role in chemotherapy resistance hasn't been reported. In this study, we found that HRD1 expression was downregulated in Tamoxifen-resistant breast cancer cell line MCF7/Tam compared to the Tamoxifen sensitive cell line MCF7. Moreover, S100A8 is the direct target of HRD1 by proteome analysis. Our data showed that HRD1 decreased the protein level of S100A8 through ubiquitination while HRD1 was regulated by acetylation of histone. More importantly, HRD1 knockdown significantly increased the cell survival of MCF7 cells to the Tamoxifen treatment. HRD1 overexpression sensitized MCF7/Tam cells to the Tamoxifen treatment *in vitro* and *in vivo*. In conclusion, the decrease of HRD1 expression contributed to Tamoxifen resistance in breast cancer.

## INTRODUCTION

The breast cancer is one of the most common cancers among the women in the whole world. Among the breast cancer, 70% are estrogen receptor alpha positive (ER+) breast cancer. Therefore, in the clinical, the major therapy is the Tamoxifen or other aromatase inhibitors in order to block estrogen receptor and inhibit the activation of downstream genes. However, about 50% of ER+ breast tumors develop resistance to these drugs including Tamoxifen [1, 2]. And thus contributes to the deaths of breast cancer patients. But, the understanding of the molecular mechanisms underlying the chemistry-drug resistance of breast cancer remains incomplete.

S100A8, a calcium-binding protein, is secreted primarily by granulocytes and monocytes, and is upregulated during the inflammatory response. It is reported that, S100A8 is associated with estrogen receptor loss in breast cancer [3]. Furthermore, S100A8 is involved in the chemistry-drug resistance in lots of kind of tumors. In leukemia cells, S100A8 promotes the autophagy so that to contribute to the drug resistance [4]. And in the breast cancer cells, the increased secretion of S100A8/A9 exacerbated the resistance of breast cancer to doxorubicin with cyclophosphamide [5]. More importantly, the CXCL1/2-S100A8/A9 loop is the cancer cell survival axis linking the chemotherapy resistance and metastasis in breast cancer and is hyperactivated by chemotherapy. Therefore, there is a possibility of clinically targeting this axis both to limit the dissemination of cancer cells and to diminish drug resistance [6].

In our recent study, we have found that HRD1, the E3 ubiquitin ligase, interacted with IGF receptor (IGF-1R) directly so that to decrease the protein level of IGF-1R through ubiquitination in both MDA-MB-231 cells and MCF7 cells. Moreover, HRD1 also took part in inhibiting the EMT in breast cancer cells [7]. Therefore, HRD1 may play as an anti-tumor role in breast cancer cells. However, whether HRD1 implicated the chemistry-drug resistance of breast cancer remains unknown. In this study, we examined the expression of HRD1 in MCF7/Tam and MCF7 cells and investigated its function in degradation of S100A8, in order to better understand its role in chemistry-drug resistance of breast cancer and its potential implications for cancer therapy.

Evidence has shown that the acetylation status of histones implicated in the chemistry-drug resistance in tumors [8–12]. The acetylation of histone promoted gene expression by increasing the approachability of promoters to the transcription machinery [13, 14]. We found that HRD1 was significantly downregulated in MCF7/Tam cells compared to MCF7 cells. We also found the histone deacetylase inhibitor, TSA, could upregulate HRD1 expression at a dose-dependent manner in MCF7 cells. We hypothesized that epigenetic modification might be involved in regulation of HRD1 expression in MCF7/Tam cells. Thus, we tested this hypothesis in both MCF7/Tam cells and MCF7 cells.

## RESULTS

### HRD1 was downregulated in MCF7/Tam cells

**Figure 1:**
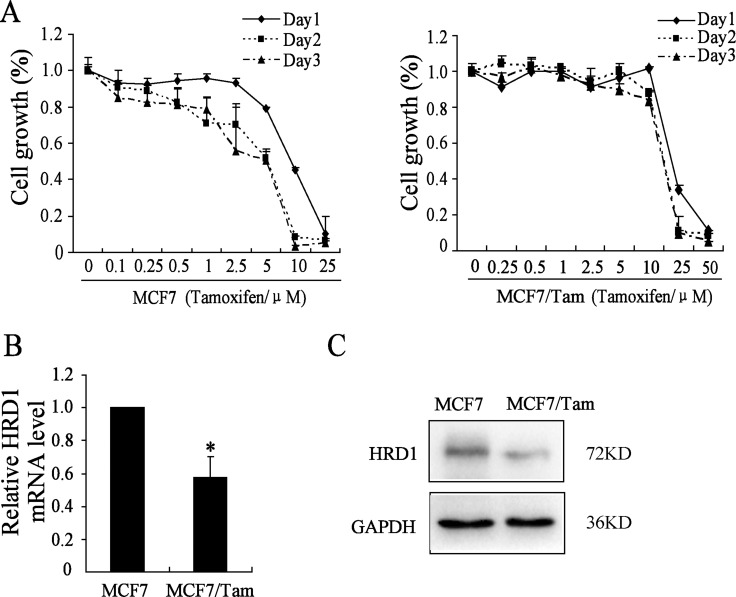
HRD1 is downregulated in MCF7/Tam cells (**A**) Cells were treated with various concentrations of Tamoxifen for about 24 h/48 h/72 h. Survived cells were measured by SRB assay. SRB assay shows that MCF7/Tam cells are much more resistant to Tamoxifen than MCF-7 cells. (**B**) Western blotting analysis showing the protein expression of HRD1 in MCF7 and Tam-R MCF7 cells. The protein level of HRD1 in MCF7 cells are much more than MCF7/Tam cell. GAPDH was used as an internal loading control. (**C**) Realtime PCR indicates a significant down-regulation of HRD1 in mRNA level in MCF7/Tam cells compared with MCF7 cells. All graphs show means ± S.D. of three independent experiments, *P < 0.05, compared to MCF7.

To better understand the biological mechanisms of chemoresistance, especially the resistance to hormone chemotherapy drugs in ER (+) breast cancer cells, we selected Tamoxifen sensitive and derived resistant breast cancer cell line pair (MCF7 and MCF7/Tam). To identify the differential sensitivity of the parental MCF7 and MCF7/Tam breast cancer cell lines to Tamoxifen, we first determined the PG (Percentage Growth) of Tamoxifen which is currently used for the treatment of ER (+) breast cancer by SRB assay. As shown in Figure 1A, MCF7/Tam cells showed resistance to Tamoxifen with higher concentrations (10 μmol/l) when the PG is 50% than MCF7 cells (5 μmol/l).

We then examined the expression of HRD1 in both protein and mRNA levels in MCF7 and MCF7/Tam cells. As shown in Figure 1B and 1C, the expression of HRD1 in MCF7/Tam cells significantly decreased in both protein and mRNA level compared to the MCF7 cells. These data suggested that, the downregulation of HRD1 may contribute to the resistance to Tamoxifen treatment.

### HRD1 directly interacted with S100A8

In order to further explore the probability of chemoresistance, we overexpressed Vector or HRD1 in MCF7 cells and then did IP assay. As shown in Supplementary Figure 1A, 23 proteins were identified with obviously changes compared between overexpression of Vector and HRD1 (ESM Table 1). Among these identified proteins, S100A8 is related to chemoresistance of breast cancers. Supplementary Figure 1B shows the relative abundance of each mass fragment in both two samples of S100A8.

**Table 1:** The results of proteome analysis

Gene Name	Peptides	Unique Peptides	Mol.Weight [kDa]	iBAQ (Ctr > 0)	iBAQ (Hrd1/Ctr)
PFKP	22	20	85.595	6578.3	1.78
PFKL	8	6	85.018	113.61	4.63
ACTG1	6	6	41.792	311.25	2.33
RPL17	2	2	19.586	69.356	1.74
RPS14	2	2	16.273	133.86	1.23
PKM	2	2	8.9512	203.02	3.53
PRDX1	2	2	18.976	220.91	2.16
S100A8	1	1	10.834	130.31	1.14
ODF1	1	1	28.366	478.03	1.18
NME1	1	1	6.5266	527.21	1.18
DUSP28	1	1	18.324	1003	1.74
LRMP	1	1	38.995	11837	1.12
LAMB1	1	1	10.082	27795	1.45
ANKFY1	6	6	128.4	149.6	0.71
EEF1A1P5	4	1	50.184	960.81	0.60
ATP5O	4	4	23.277	1133.8	0.60
RPS18	3	3	17.718	270.06	0.42
CFL1	2	2	10.181	379.28	0.80
RPS25	2	2	13.742	635.14	0.34
DCD	2	2	11.284	723.04	0.31
RPS20	2	2	13.373	962.15	0.51
HIST1H4A	1	1	11.367	574.18	0.69
MPI	1	1	19.385	873.03	0.71

**Figure 2:**
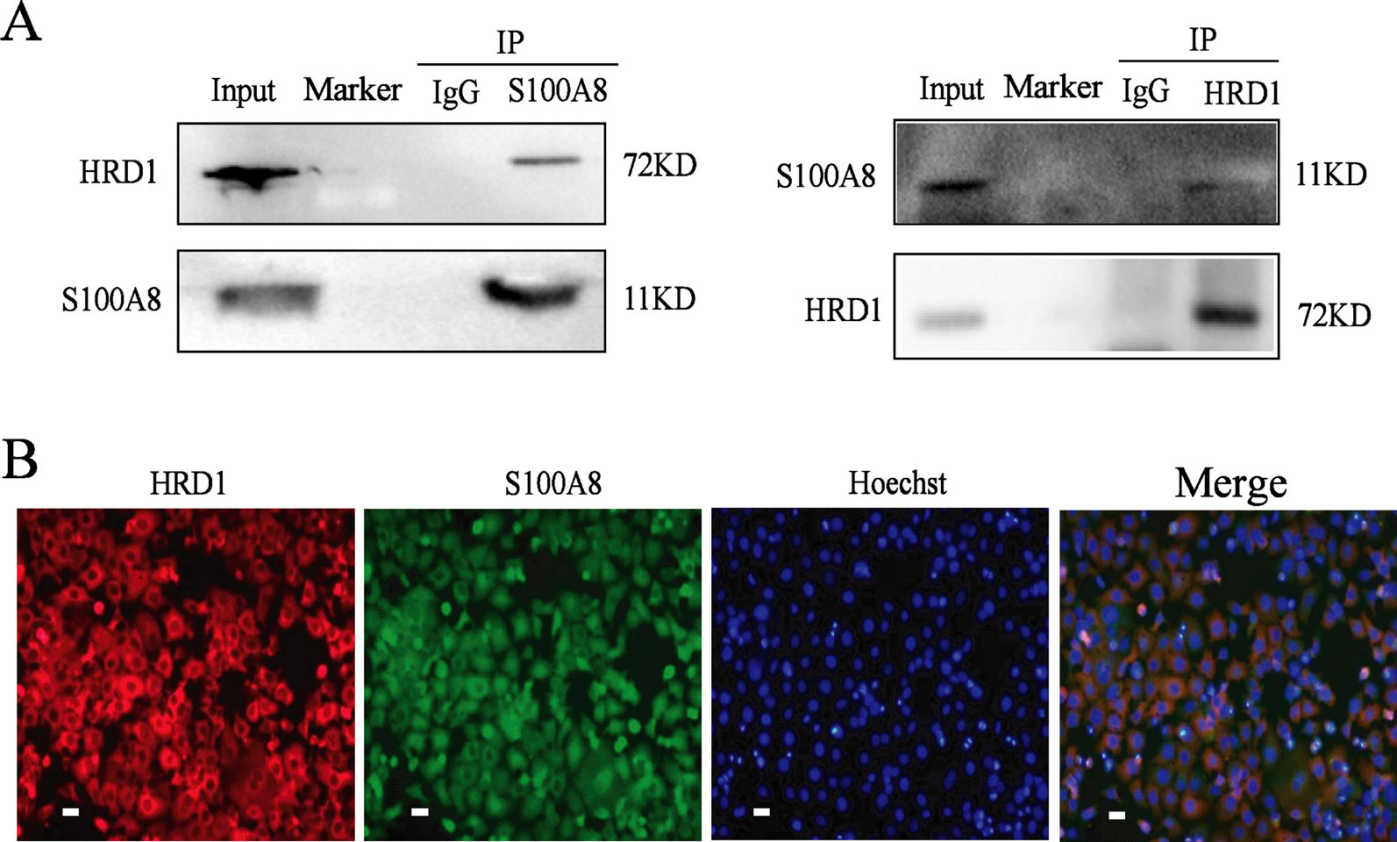
HRD1 directly interacted with S100A8 (**A**) MCF-7 cells were pretreated with MG132 (10 μmol/l) for 6 h and endogenous protein-protein interactions between HRD1 and S100A8 were determined by immunoprecipitation (IP) with HRD1 or S100A8 antibodies, followed by immunoblotting. IgG was used as a negative control for IP. (**B**) Inmmunofluorescence to certificate HRD1 and S100A8 can co-locate in the cytoplasm in MCF7 cells (Scale bar = 20 μm).

To verify the results from proteome analysis, we then did the Co-IP. As shown in Figure 2A, HRD1 interacted with S100A8 in MCF7 cells by Co-IP. Furthermore, the results of immunofluorescence show that, HRD1 and S100A8 co-located in the cytoplasm of the breast cancer cells (Figure 2B).

### HRD1 promoted the degradation of S100A8 through ubiquitination

**Figure 3:**
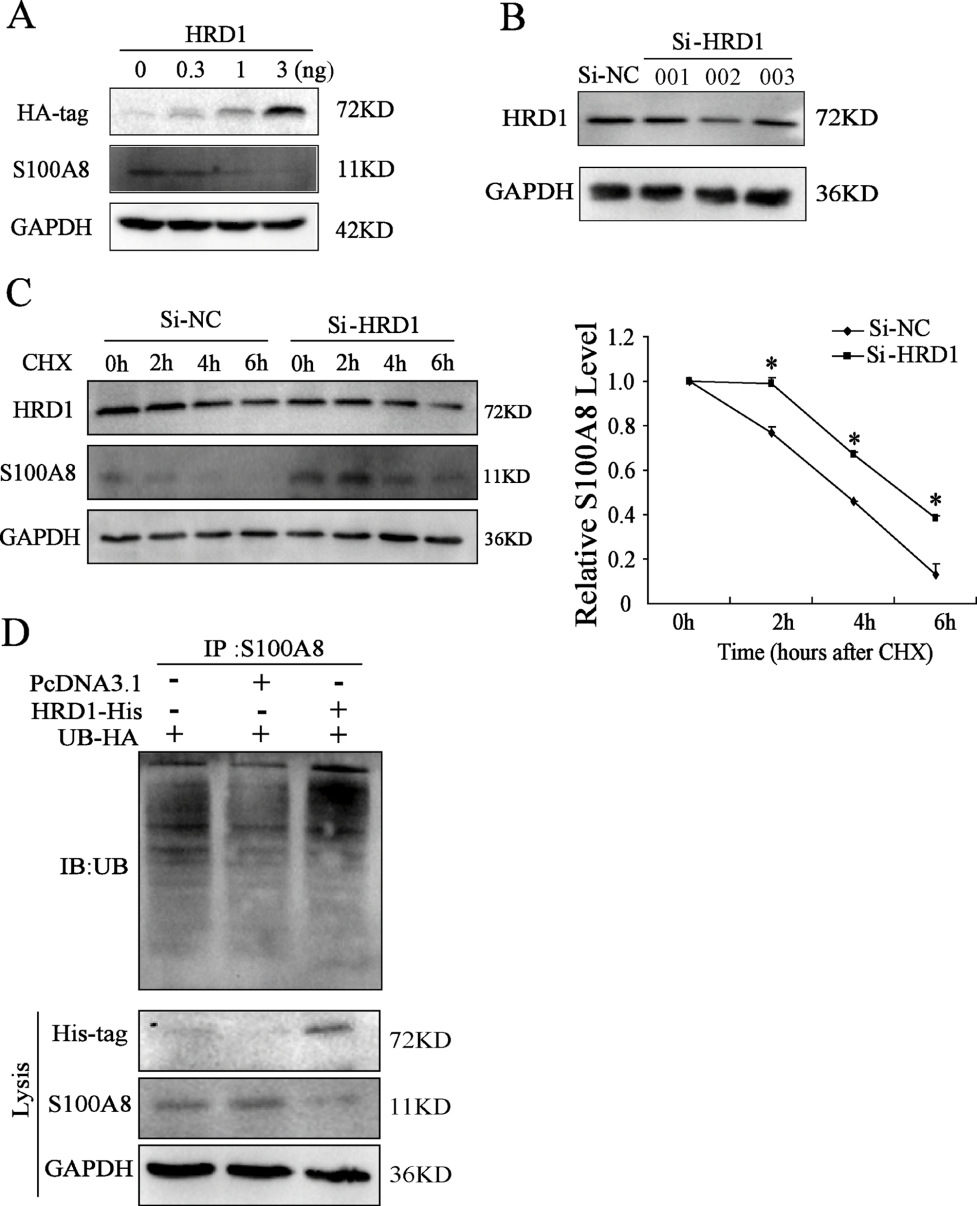
HRD1 promotes the degradation of S100A8 through ubiquitination (**A**) The protein level of HRD1, S100A8 through Western blotting analysis in MCF7 cells with overexpression of different dose of HRD1. (**B**) The protein level of HRD1 in MCF7 cells by knockdown HRD1 through Western blotting analysis. (**C**) MCF7 cells were transfected with si-HRD1 and the control for 48 h, followed by exposure to cycloheximide (CHX 50ng/ml) for 0, 2, 4, 6 h. The protein of S100A8 and HRD1 in whole cell lysates was measured. (**D**) Ubiquitination of S100A8 was induced by HRD1. HA-ubiquitin was co-expressed in MCF7 cells with His-HRD1 or Vector control with treatment of MG132 (10 μmol/l) for 4 h. Ubiquitinated S100A8 was immunoprecipitated using S100A8 antibody and further detected with UB antibody. The endogenous S100A8 and His-HRD1 in the whole cell lysates were examined by S100A8 and His-tag antibodies. *P < 0.05, compared to Si-NC.

In MCF7 cells, overexpression of HRD1 inhibited S100A8 protein level at a dose-dependent manner (Figure 3A). For HRD1 is an E3 ubiquitin ligase, we further explored the potential mechanisms related to the decrease of HRD1 on S100A8. We treated MCF7 cells with cycloheximide (CHX), an inhibitor of protein synthesis, resulted in promotion of S100A8 degradation by overexpressing HRD1 (Supplementary Figure 2A). However, this degradation was suppresses by HRD1 knockdown (Figure 3C).

Moreover, an elevation in ubiquitinated S100A8 was found. As shown in Figure 3D, when inhibited the degradation of S100A8 by MG132, overexpression of HRD1 further increased the ubiquitination of S100A8. Therefore, these results indicated that HRD1 served as an E3 ubiquitin ligase so that to promote the ubiquitination of S100A8 to degrade by the proteasome.

### HRD1 was upregulated by acetylation of histone

**Figure 4:**
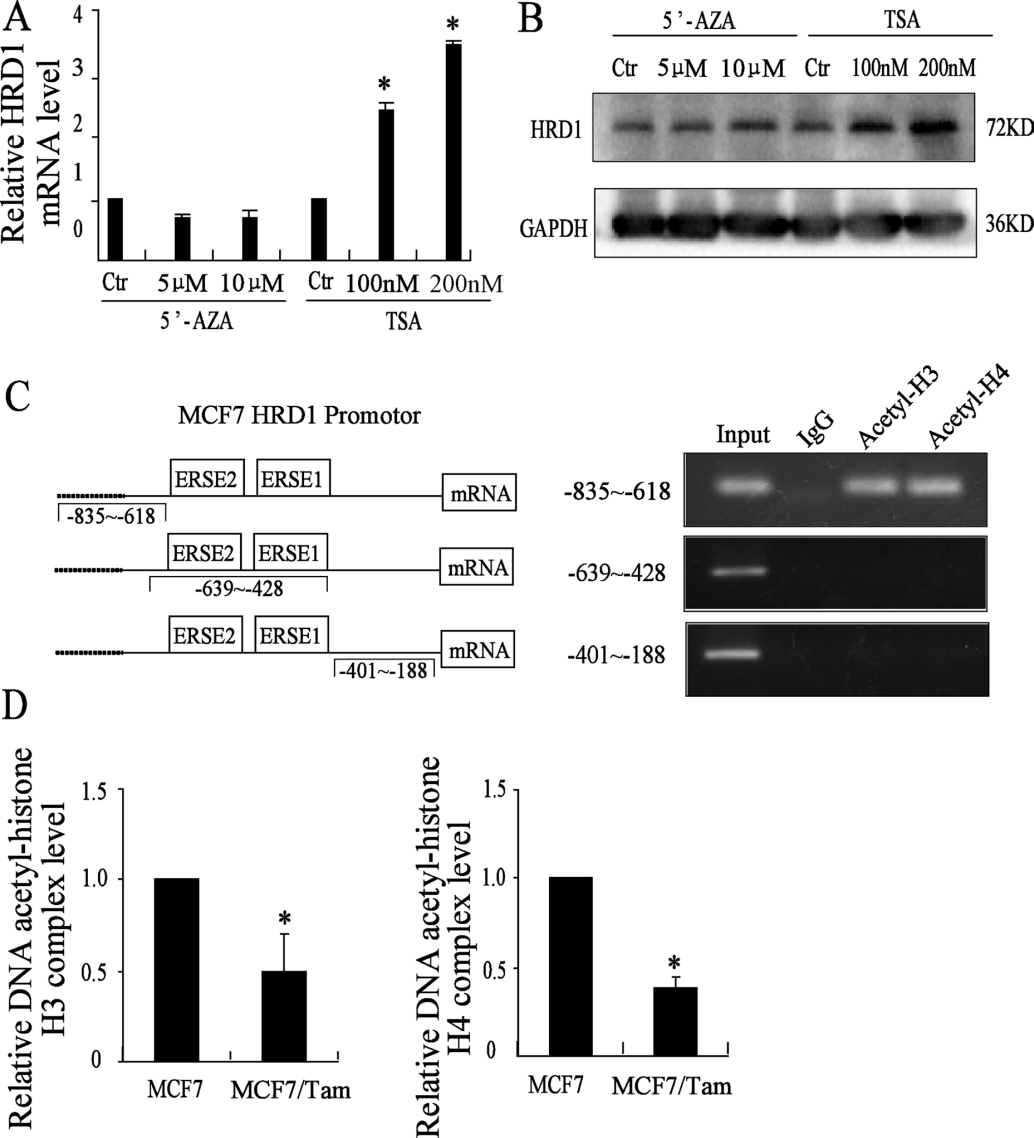
HRD1 is upregulated by acetylation of histone (**A**) Different concentration of 5′AZA and TSA used in the MCF7 cells for 72 h, expression of HRD1 protein level determined by Western blotting. (**B**) Different concentration of 5′AZA and TSA used in the MCF7 cells for 72 h, expression of HRD1 mRNA level determined by qRT-PCR. (**C**) The HRD1 promotor region mode pattern on the left. On the right, CHIP assay did with anti acetyl-histone H3 and H4 and negative control anti-IgG (Rabbit). A agarose gel (1%) of PCR products amplified with primers to the region of HRD1 promotor respectively (−835~−618, −639~−428, −401~−188). (**D**) qRT-PCR tests the Dna-Acetyl-histone H3 and H4 binding levels in both MCF7 cells and MCF7/Tam cells. All graphs show means ± S.D. of three independent experiments, *P < 0.05, compared to MCF7.

To determine whether the expression of HRD1 is regulated by methylation of DNA or by acetylation of histone, MCF7 cells were treated with different concentration of 5′-AZA, a DNA methyltransferases inhibitor and TSA, a histone deacetylases inhibitor for about 72 h, followed by real-time PCR assay and Western blotting. The results showed that the treatment of 5′-AZA didn't change the expression of HRD1 at both mRNA and protein level. However, the treatment of TSA upregulated the expression of HRD1 at both mRNA and protein level at a dose-dependent manner compared to the control cells (Figure 4A, 4B). These results indicated that HRD1 was regulated by acetylation of histone. However, we also tested the expression of S100A8 at both mRNA and protein level by the treatment of TSA as well as 5′-AZA. The result showed hat the treatment of TSA can increase the mRNA expression of S100A8 at a dose-dependent manner while 5′-AZA cannot. But, the protein expression of S100A8 didn't change. It may due to both the increased mRNA expression of S100A8 and the increased protein expression of HRD1 (Supplementary Figure 3).

Next, we did ChIP-qPCR assay to test whether the downregulation of HRD1 in MCF7/Tam cells is due to the downregulation of acetylation of histone in the HRD1 promotor region. The results showed that acetyl-histone H3 and acetyl-histone H4 could bind to the HRD1 promotor region (Figure 4C). Moreover, we found that the acetylation of histone H3 and histone H4 in the promoter region in MCF7/Tam cells were downregulated compared to MCF7 cells (Figure 4D). These results suggested that HRD1 was regulated by acetylation of H3 and H4 histone.

### HRD1 was involved in breast cancer resistance by directly targeting S100A8

**Figure 5:**
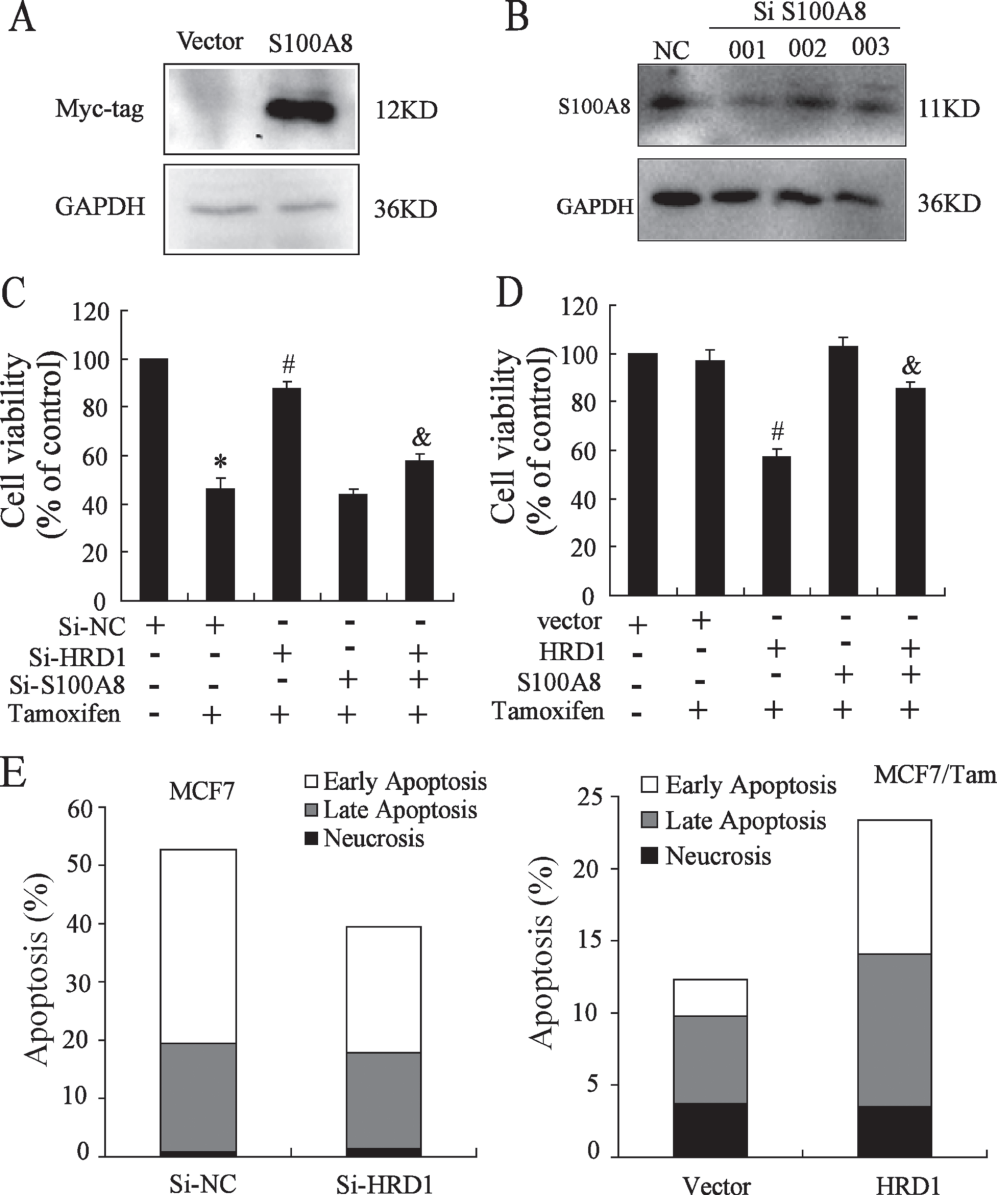
HRD1 can sensitize MCF-7 and MCF7/Tam cells to Tamoxifen (**A**) S100A8 plasmid is constructed and tested by Western blotting. (**B**) Knockdown of S100A8 in MCF7 cells and tested by Western Blot. (**C**) MCF7 cells were transfected SiNC, SiHRD1, SiS100A8 and co-transfected SiHRD1 and SiS100A8 respectively, followed by treatment of 5 μM Tamoxifen. After 48 h, SRB assay played. (**D**) MCF7/Tam cells were transfected Vector, HRD1, S100A8 and co-transfected HRD1 and S100A8 respectively, followed by treatment of 10 μmol/l Tamoxifen. After 48 h, SRB assay played. (**E**) MCF7 cells were transfected SiNC and SiHRD1 respectively for 24 h followed by treatment of 5 μmol/l Tamoxifen. After 48 h, the flow cytometry played. (**F**) MCF7/Tam cells were transfected Vector and HRD1 plasmid respectively for 24 h followed by treatment of 10 μmol/l Tamoxifen. After 48 h, the flow cytometry was performed. All graphs show means ± S.D. of three independent experiments. **P < 0.05, compare to Si-NC or vector; ^#^*P* < 0.05, compare to Si-NC or vector + Tamoxifen; ^&^*P* < 0.05, compare to Si-HRD1 or HRD1 + Tamoxifen.

To further investigate the association of HRD1 expression with breast cancer chemoresistance, we constructed S100A8 plasmid and chose the SiRNA of S100A8 (Figure 5A, 5B). Because of the expression of HRD1 in MCF7 cells was higher than MCF7/Tam cells, we transfected Si-NC, Si-HRD1 and/or Si-S100A8 in MCF7 cells while transfected vector, HRD1 and/or S100A8 in MCF7/Tam cells, followed by treatment of 5 μmol/l Tamoxifen in MCF7 cells and 10 μmol/l Tamoxifen in MCF7/Tam cells. The inhibition of cell proliferation by the Tamoxifen was obviously decreased by knockdown of HRD1 in MCF7 cells, which was reversed by S100A8 deletion (Figure 5C). Meanwhile, the inhibition of cell proliferation by the Tamoxifen was significantly increased by overexpression of HRD1 in MCF7/Tam cells, which was reversed by overexpression of S100A8 (Figure 5D). Moreover, the results of flow cytometry showed that knockdown of HRD1 in MCF7 cells reduced the apoptosis rate of cells with the treatment of 5 μmol/l Tamoxifen while overexpression of HRD1 in MCF7/Tam cells increased the apoptosis rate of cells with the treatment of 10 μmol/l Tamoxifen (Figure 5E–5G, Supplementary Figure 4A). These results indicated that overexpression of HRD1 could sensitize MCF7/Tam cells to the treatment of Tamoxifen while knockdown of HRD1 can be resistant MCF7 cells to Tamoxifen.

### Overexpression of HRD1 increased the sensitivity of drug-resistant breast tumors to tamoxifen treatment *in vivo*

**Figure 6:**
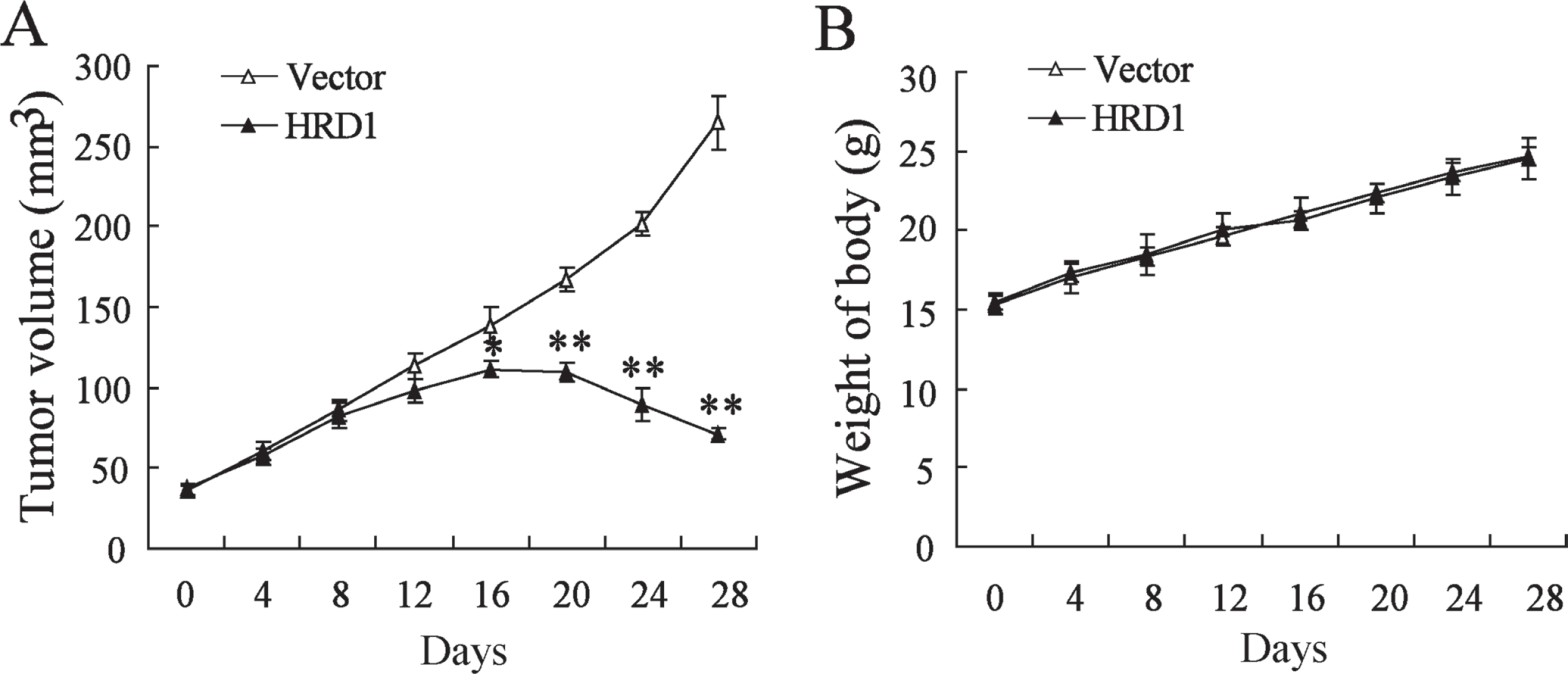
Overexpression of HRD1 increased the sensitivity of drug-resistant breast tumors to Tamoxifen treatment *in vivo* MCF7/Tam cells stably overexpressing of HRD1 and the corresponding controls were injected into the right flank and left flank of nude mice, respectively. Tumor volume (**A**) and body weights of mice with tumors (**B**) were measured. *P < 0.05, compared to vector.

We explored the *in vivo* effects of HRD1 on the breast cancer chemoresistance by injecting MCF7/Tam cells stable overexpressing HRD1 or the corresponding controls into nude mice, respectively (Supplementary Figure 5). As expected, overexpresssion of HRD1 significantly increased the sensitivity of MCF7/Tam cells to Tamoxifen. As indicated in Figure 6A, the growth of xenograft tumors was much slower in the group transfected with EGFP-tagged HRD1 lentivirus than in the group transfected with EGFP lentivirus. However, the body weight did not exhibit obvious difference between the two groups (Figure 6B).

## DISCUSSION

In nowadays, breast cancer becomes one of the most common cancers among the women in the worldwide. However, about 50% ER+ breast cancer will be onset of resistance as well as recurrence after the treatment of Tamoxifen and other aromatase inhibitors [1, 2]. Therefore, the breast cancer is still the major killer for the women in the whole world. In our study, we have found the E3 ubiquitin ligase of HRD1 was involved in the Tamoxifen resistance of breast cancer and could be the new target for the treatment of ER+ breast cancer in the clinical.

Acetylation status of histones has been shown to be involved in the drug resistance in breast cancer. Histone decetylase 4 (HDAC4) could mediate the deacetylation of SMAD family 4 so that induce the 5-fluorouracil resistance in both ER+ and ER- breast cancer cells [15]. Moreover, in ER+ breast cancer cells, the resistance to fulvestrant was due to the modulated expression of GPER and CDK6 in which the deacetylase was implicated [16]. In this study, we found that the histone deacetylase inhibitor (TSA) could upregulate HRD1 expression in breast cancer cells. However, the specific inhibitor of DNA methylation (5-Aza-CdR) had no effect on HRD1 expression. These results indicated that HRD1 is regulated by acetylation of histone in breast cancer cells. Further studies using ChIP analysis confirmed that H4 and H3 histone bound to the promoter region of HRD1 (−835 to −618), which is adjoined to two ER stress response elements (ERSE1 and ERSE2) [17–18]. Further investigation of correlation between acetylation of histone and ERSE will be benefit to understand how HRD1-mediated drug resistance in breast cancer in future.

It is also important to recognize that acetylation of histone H3 and H4 in HRD1 promoter was significantly decreased in MCF7/Tam cells. These results clearly demonstrate that the lower acetylation of histone resulted in the lower expression of HRD1 in MCF7/Tam cells compared to MCF7 cell. Meanwhile, overexpression of HRD1 in MCF7/Tam cells increased the response to Tamoxifen, while inhibition of HRD1 in MCF7 cells decreased the response to Tamoxifen. More importantly, HRD1 overexpression increased the sensitivity of drug-resistant breast tumors to Tamoxifen treatment in animal model. These results indicated that HRD1 mediated chemotherapy resistance of breast cancer.

We identified for the first time S100A8 as the down-stream target of HRD1 in mediating its drug-resistant effects on breast cancer cells. S100A8 plays an important role in chemotherapy resistance and metastasis of breast cancer [3, 6]. In the present study, we found that HRD1 downregulated S100A8 expression. Furthermore, we demonstrated that HRD1 could interact with S100A8 acting as a ubiquitin E3 ligase that targeted S100A8 for degradation through proteasomal degradation pathway. In MCF7 cells, S100A8 deletion reversed the effect of knockdown of HRD1 on Tamoxifen treatment. In MCF7/Tam cells, S100A8 overexpression reversed the effect of upregulation of HRD1 on Tamoxifen treatment. Therefore, HRD1 mediated breast cancer resistance by directly inhibiting S100A8 expression.

In conclusion, our findings indicated that HRD1 expression was negatively correlated with the Tamoxifen resistance of ER+ breast cancers. The lower acetylation of histone was responsible for the downregulation of HRD1 in breast cancer cells. Overexpression of HRD1 increased the response of breast cancer to Tamoxifen by inhibiting S100A8 expression. Based on our findings, we proposed that restoration of HRD1 expression may be an improved strategy for endocrine therapy for human ER+ breast cancers.

## MATERIALS AND METHODS

### Cell lines and culture

The Tamoxifen resistance human breast cancer MCF7/Tam cells and Tamoxifen sensitive human breast cancer MCF7 cells were gifts from Doc. Chen (Nanjing Southeast University). The MCF7/Tam cells and MCF7 cells were cultured in RPMI-1640 (Invitrogen, Carlsbad, CA) supplemented with 10% Fetal Bovine Serum (FBS) and were added with the indicated 1μmol/l Tamoxifen for resistance maintenance. All cells were cultured at 37°C in a humidified atmosphere containing 95% air and 5% CO2 [19].

### Cell transfection

MCF7 and MCF7/Tam cells were transfected with different amount of HRD1 and S100A8 or the vector pCMV5-myc using Lipofectamine 2000, according to the manufacturer's instruction. After transfection for 24 h, cells were then treated with different concentration of Tamoxifen, and then cells were used for Western blot and qRT-PCR.

### RNAi plasmid and plasmid construction

HRD1 and S100A8 expression was silenced utilizing specific small interfering RNA (HRD1-siRNA, S100A8-siRNA and Si-NC) purchased from Genechem (Shanghai, China). HRD1-siRNA and Si-NC were the same as described in our previous study [7]. And the sequence of S100A8-siRNA wAS on ESM Table 1. The S100A8 expression plasmid was constructed by inserting the full-length coding region sequences into pCMV5-myc vector between NdeI and BamHI. The primer sequence of the full-length coding region of S100A8 is: 5′-GGAATTCCATATGATGTTGAC CGAGCTGGAG-3′ (Forward), 5′-CGGGATCCCTACTC TTTGTGGCTTTCTTC-3′ (Reverse). The HRD1 expression plasmid was described previously [7]. All constructions used here were sequenced and confirmed to be correct.

### Co-immunoprecipitation (Co-IP) and proteome analysis

Co-immunoprecipitation (Co-IP) was performed according to our previous methods [7]. And after using SDS-PAGE to test the IP samples staining by CBB (Coomassie Brilliant Blue), we then cleaved the two whole sample lanes to submit the cleaved lanes to Analysis and Test Centre of Nanjing Medical University to do the proteome analysis.

### SRB assay for cell survival

MCF7 and MCF7/Tam cells were seeded onto 96-well plates at a density of 1,000 cells/well. After culture for 24 h, cells were transfected with siRNA or overexpression plasmid 24 h. Then, MCF7 cells were treated with 5 μmol/l Tamoxifen and MCF7/Tam cells were treated with 10 μmol/l Tamoxifen for 48 h. Next, cells were treated with 10% TCA (w/v) for 12 h. And then, 0.4% SRB (Sigma, USA) was added for about 30min. With 10 μmol/l PH = 10.5 Tris-base, the absorbance at 540 nm was measured using a multi-mode reader (LD942, Beijing, China). Besides, MCF7 and MCF7/Tam cells were seeded onto 96-well plates at a density of 1,000 cells/well. After culture for 24 h, cells were treated with serial dilutions of Tamoxifen for 48 h, followed by SRB Assay. The IC50 (50% inhibitory concentration) value was calculated by normal probability transforms according to the relationship of drug concentration and inhibition rate.

### Real-time PCR assay

Real-time PCR assay was performed to detect the relative expression of mRNA. Briefly, total RNA was isolated from MCF7 and MCF7/Tam cells using Trizol reagents (Invitrogen, CA, USA) according to the manufacture's protocol. RNA was reverse transcribed into complementary DNA using ReverTra ACE (Toyobo, Shanhai, China). Real-time PCR assay was performed using SYBR Premix Ex Taq™ II kit (Takara, Dalian, China) in a final volume of 10 μl mixture containing 1 μl cDNA, 0.5μl of each primer, and 5 μl SYBRGreen. Relative mRNA expression was normalized to GAPDH expression. Sequences of the primers used were available in ESM Table 1.

### Western blotting

MCF7 and MCF7/Tam cells were washed with ice-cold PBS for 2 times, and then were added with RIPA lysis buffer. Protein in each sample was analyzed by western blotting as previously reported [7]. The dilution of each antibodies is: anti-S100A8 (1:500) (Proteintech, Chicago, USA), anti-HRD1 (1:1000) (Abcam, Cambridge, UK), anti-GAPDH (1:4000) (Abcam, Cambridge, UK), anti-HA-tag (1:1000) (Proteintech, Chicago, USA), anti-His-tag (1:1000) (Santz Cruz, CA, USA), anti-Myc-tag (1:1000) (Proteintech, Chicago, USA).

### Flow cytometry

Flow cytometry was performed on a Guava easyCyte HT System Flow Cytometer (Millipore) using Annexin V-FITC Apoptosis Detection Kit (Vazyme, Nanjing, China) for analysis of early and late apoptotic cells according to the manufacturer's instructions (Vazyme).

### Chromatin immunoprecipitation (ChIP) assay and ChIP-qPCR assay

ChIP assays were performed using a commercially available ChIP Assay Kit (Millipore) according to our previous methods [7]. Sequences of the primers used were available in ESM Table 2.

**Table 2:** Sequences of oligonucleotides used as primers for real-time PCR, siRNA and ChIP-qPCR

Construct		Sequences
Primers for real-time PCR		
HRD1	Sense	5′-AACCCCTGGGACAACAAG-3′
	Antisense	5′-GCGAGACATGATGGCATCT-3′
S100A8	Sense	5′-ATGCCGTCTACAGGGATGAC-3′
	Antisense	5′-ACTGAGGACACTCGGTCTCT-3′
GAPDH	Sense	5′-ATGGGGAAGGTGAAGGTCG-3′
	Antisense	5′-GGGGTCATTGATGGCAACAATA-3′
Primers for siRNA		
Si S100A8 001	Sense	5′-CUAUCAUCGACGUCUACCATT-3′
	Antisense	5′-UGGUAGACGUCGAUGAUAGTT-3′
Si S100A8 002	Sense	5′-GGAAUUUCCAUGCCGUCUATT-3′
	Antisense	5′-UAGACGGCAUGGAAAUUCCTT-3′
Si S100A8 003	Sense	5′-GACGUCUGGUUCAAAGAGUTT-3′
	Antisense	5′-ACUCUUUGAACCAGACGUCTT-3′
Primers for CHIP-qPCR		
−835~−618	Sense	5′-AAGCAGGTGAGTGGTTGTTAGGG-3′
	Antisense	5′-ACAAACAGAACACTTCCGTCCC-3′
−639~−428	Sense	5′-CAACAGTTTGACAAATACGGTGC-3′
	Antisense	5′-CCTAACAACCACTCACCTGCTTT-3′
−401~−188	Sense	5′-GGTGGTCGGAAGGTAATAAGGC-3′
	Antisense	5′-CATTTCATTCCCCTCAGGCTTGT-3′

### Immunofluorescence

Fifty percent confluent cells were cultured on glass coverslips in the wells of 24-well plates for 24h. Then cells were washed, fixed, and permeabilized with 0.3% Triton X-100 for 10 min. After blocking with goat serum for 2 h, cells were incubated for 1 h with antibody anti-S100A8 (1:30dillution) (Proteintech, Chicago, USA) and Anti-HRD1 (1:50 dilution) (Abcam, Cambridge, UK). Then, dishes were washed and incubated with Alexa Fluor 488 or Alexa Fluor 594-conjugated secondary antibodies (1:50 dilution) for 1h at room temperature. Nuclei were stained with Hoechst (10 mg/ml) for 2 min. Samples were examined with fluorescence microscope (Zeiss, Oberkochen, German).

### Animal tumor model

Female athymic nude mice (6-weeks-old) were purchased from Shanghai Laboratory Animal Centre (Chinese Academy of Sciences, Shanghai, China) and maintained in cage housing under specific pathogen-free conditions. Cultured MCF7/Tam cells were transfected with EGFP or EGFP-tagged HRD1 lentivirus according to previous report [7]. Cells were injected either into the right or left flank region of the mice to generate subcutaneous xenografts. Tamoxifen was given once the size of the xenograft reached approximately 4 mm in diameter. The mice were randomly assigned into two groups of eight. They were treated with Tamoxifen by intragastric administration with dosage of 25 mg/kg (twice a day) for 2 weeks. Tumor volumes were estimated using the formula: 0.5 × length × width^2^. This study was carried out in accordance with the guidelines of the Institutional Animal Care and Use Committee at Nanjing Medical University and was approved by the Committee on the Ethics of Animal Experiments of Nanjing Medical University.

### Statistical analysis

Data were analyzed using the SPSS statistics 16.0 software package. Comparisons were performed using the Student's *t* test between two groups or ANOVA in multiple groups. A *P* value < 0.05 was considered to be statistically significant.

## SUPPLEMENTARY MATERIALS FIGURES AND TABLES


