# Supplementary material for: HRD1 sensitizes breast cancer cells to Tamoxifen by promoting S100A8 degradation
Source: Oncotarget. 2017 Mar 1;8(14):23564–74. doi: 10.18632/oncotarget.15797 (PMC5410327; doi:10.18632/oncotarget.15797)
Supplement: Supplementary file 1 [file oncotarget-08-23564-s001.pdf]

HRD1 sensitizes breast cancer cells to Tamoxifen by promoting S100A8 degradation

Supplementary Materials

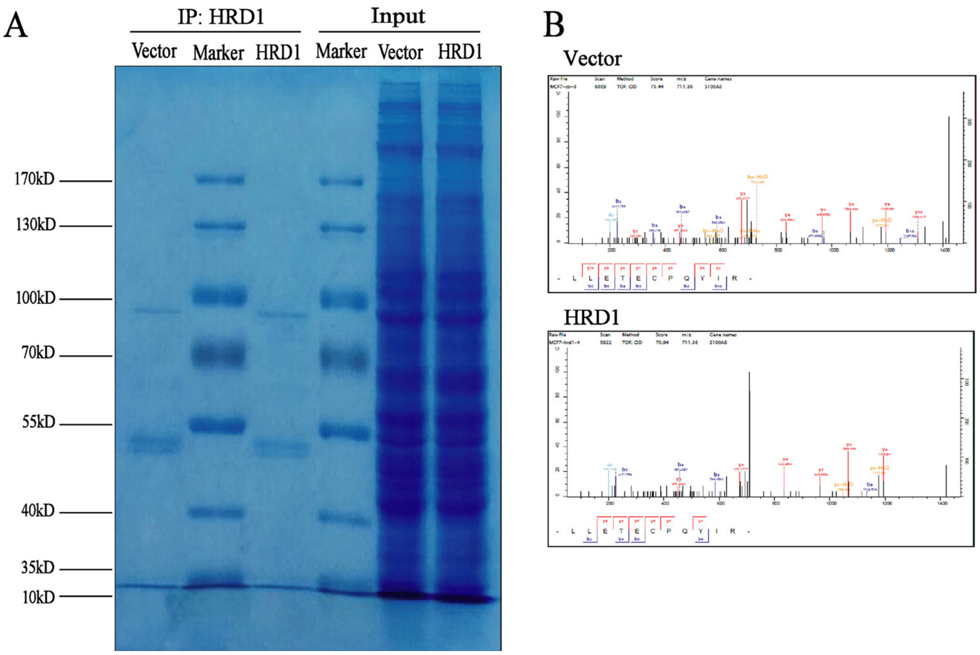

Supplementary Figure 1: HRD1 directly interacted with S100A8. (**A**) Vector or HRD1 was overexpressed in MCF7 cells respectively. SDS-PAGE to test the inmmunoprecipitated samples by HRD1 antibodies and the whole gel was stained by CBB (Coomassie Brilliant Blue). (**B**) The relative abundance of each mass fragment in both two samples of S100A8.

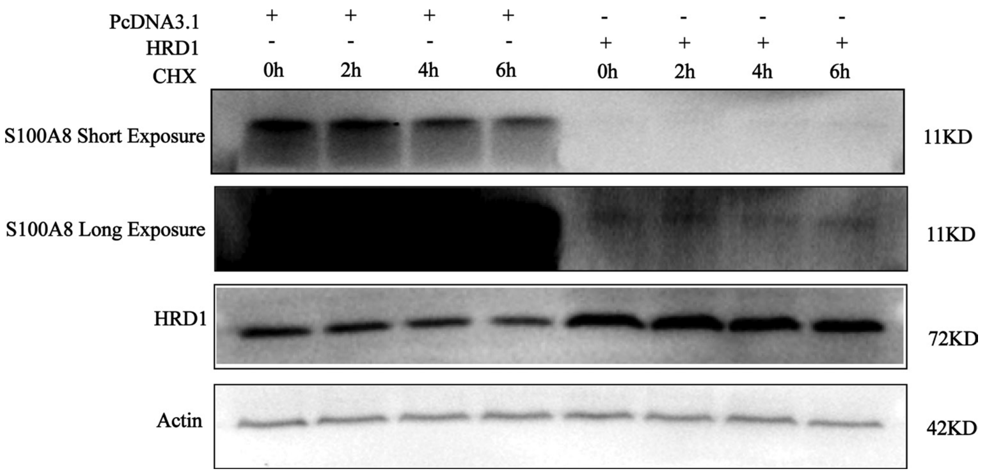

Supplementary Figure 2: HRD1 promoted the degradation of S100A8 through ubiquitination. (**A**) MCF7 cells were transfected with overexpression of HRD1 and the control for 24 h, followed by exposure to cycloheximide (CHX 50 ng/ml) for 0, 2, 4, 6 h. The protein of S100A8 and HRD1 in whole cell lysates was measured.

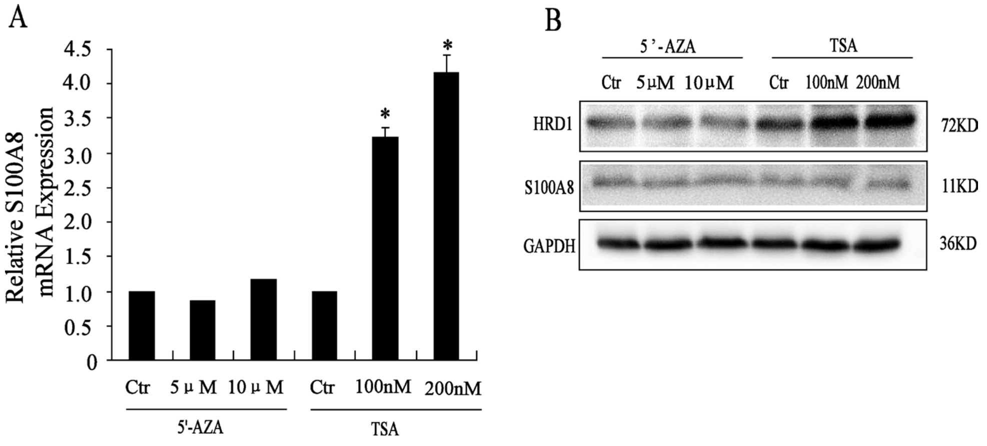

Supplementary Figure 3: HRD1 was upregulated by acetylation of histone. (**A**) Different concentration of 5′AZA and TSA used in the MCF7 cells for 72 h, expression of S100A8 mRNA level determined by qRT-PCR. (**B**) Different concentration of 5′AZA and TSA used in the MCF7 cells for 72 h, expression of S100A8 and HRD1 protein level determined by Western blotting. All graphs show means ± S.D. of three independent experiments, *$P < 0.05$, compared to the control.

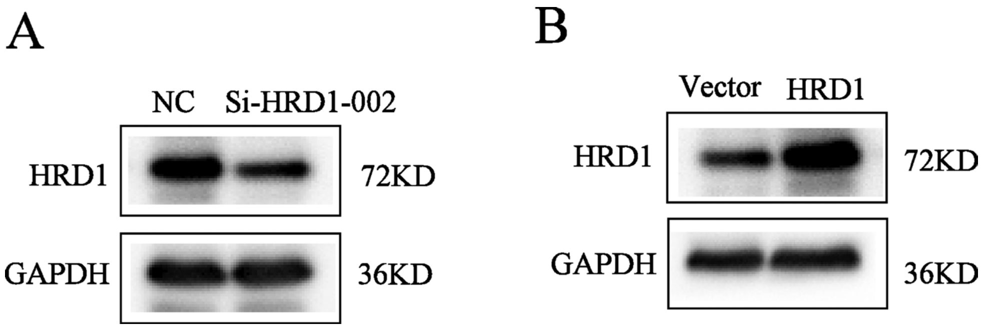

Supplementary Figure 4: HRD1 can sensitize MCF-7 and MCF7/Tam cells to Tamoxifen. (**A**) Knockdown of HRD1 in MCF7 cells and tested by Western blotting. (**B**) HRD1 plasmid is constructed and tested in MCF7/Tam cells by Western blotting.

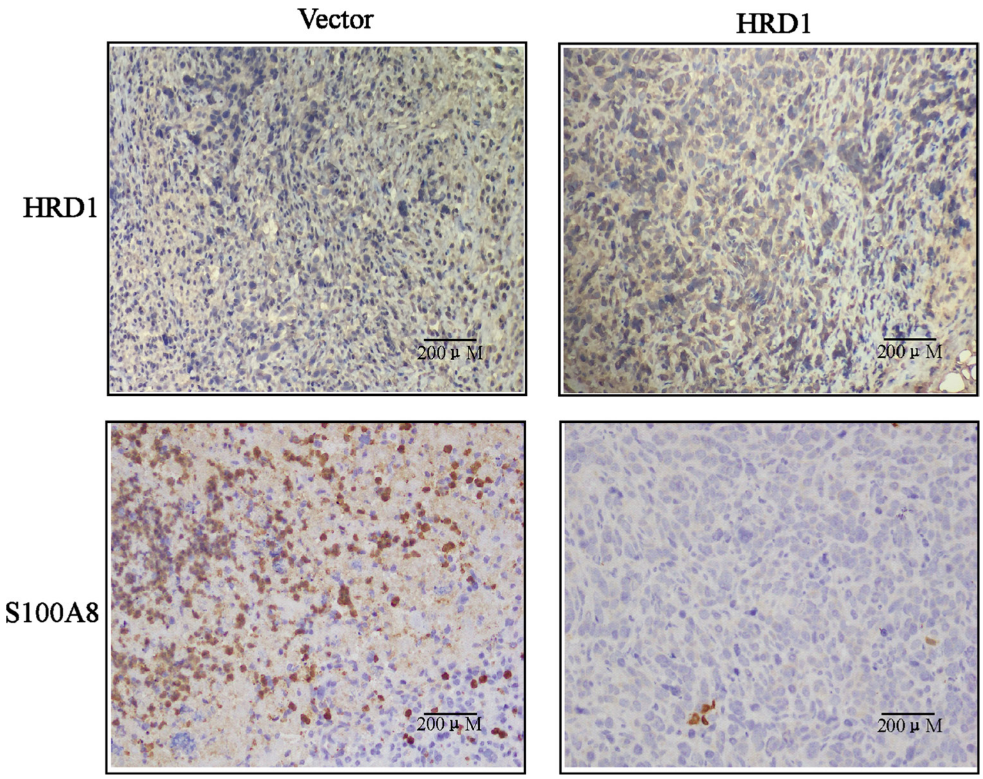

Supplementary Figure 5: Overexpression of HRD1 increased the sensitivity of drug-resistant breast tumors to Tamoxifen treatment *in vivo.* MCF7/Tam cells stably overexpressing of HRD1 were injected into the right flank and left flank of nude mice, respectively After 4 weeks, the xenograft tumors from mice in each experimental group were examined for the expression of both HRD1 and S100A8 by immunohistochemistry.
